# End of life decision making when home mechanical ventilation is used to sustain breathing in Motor Neurone Disease: patient and family perspectives

**DOI:** 10.1186/s12904-024-01443-1

**Published:** 2024-05-02

**Authors:** Eleanor Wilson, Jonathan Palmer, Alison Armstrong, Ben Messer, Edward Presswood, Christina Faull

**Affiliations:** 1 School of Health Sciences, University of Nottingham, Nottingham, UK; 2 University Hospitals, University Hospitals NHS Trust Plymouth, Plymouth, UK; 3 Newcastle upon Tyne Hospitals NHS Foundation Trust, Newcastle upon Tyne, UK; 4 Dorothy House Hospice Care, Bath, Newcastle upon Tyne, UK; 5 LOROS , Hospice, Leicester, UK

**Keywords:** Motor neurone disease (MND), Decision-making, Non-invasive ventilation (NIV), Home mechanical ventilation (HMV), End-of-life, Withdrawal of treatment, Interviews, Patient perspectives, Family perspectives

## Abstract

**Background:**

Motor Neurone Disease (MND) leads to muscle weakening, affecting movement, speech, and breathing. Home mechanical ventilation, particularly non-invasive ventilation (NIV), is used to alleviate symptoms and support breathing in people living with MND. While home mechanical ventilation can alleviate symptoms and improve survival, it does not slow the progression of MND. This study addresses gaps in understanding end-of-life decision-making in those dependent on home mechanical ventilation, considering the perspectives of patients, family members, and bereaved families.

**Methods:**

A UK-wide qualitative study using flexible interviews to explore the experiences of people living with MND (*n* = 16), their family members (*n* = 10), and bereaved family members (*n* = 36) about the use of home mechanical ventilation at the end of life.

**Results:**

Some participants expressed a reluctance to discuss end-of-life decisions, often framed as a desire to “live for the day” due to the considerable uncertainty faced by those with MND. Participants who avoided end-of-life discussions often engaged in ‘selective decision-making’ related to personal planning, involving practical and emotional preparations. Many faced challenges in hypothesising about future decisions given the unpredictability of the disease, opting to make ‘timely decisions’ as and when needed. For those who became dependent on ventilation and did not want to discuss end of life, decisions were often ‘defaulted’ to others, especially once capacity was lost. ‘Proactive decisions’, including advance care planning and withdrawal of treatment, were found to empower some patients, providing a sense of control over the timing of their death. A significant proportion lacked a clear understanding of the dying process and available options.

**Conclusions:**

The study highlights the complexity and evolution of decision-making, often influenced by the dynamic and uncertain nature of MND. The study emphasises the need for a nuanced understanding of decision-making in the context of MND.

**Supplementary Information:**

The online version contains supplementary material available at 10.1186/s12904-024-01443-1.

## Background

Motor Neurone Disease (MND), including Amyotrophic Lateral Sclerosis (ALS), causes muscles to weaken, resulting in difficulties in movement, speech, swallowing and breathing. It can also result in cognitive impairments for 20–40% of people living with MND (plwMND) [1]. When breathing is affected, plwMND can choose to use home mechanical ventilation (HMV) to palliate symptoms, support breathing, and improve quality of life and survival [1, 2]. In the UK, HMV is predominantly in the form of non-invasive ventilation (NIV), where breathing is supported using a face or nasal mask interface. Less than 1% of patients have invasive ventilation via a tracheostomy [3]. Approximately 60% of plwMND opt for a trial of HMV, with 83% of these successfully adapting to it [3] and 23% becoming full dependent with 24 h use [4]. Whilst using HMV may improve symptoms and prolong survival it does not affect the neuro-muscular progression. Patients will eventually become totally dependent for all aspects of living and can lose the ability to communicate, entering a so-called ‘locked-in’ state. MND has no cure and the majority of deaths are from respiratory failure [5].

In line with implementation of NICE guidance [6], there is a growing need to support discussions about the use and the cessation of HMV. NICE recommends that discussions about the wider context of palliative care, end of life decisions, and potential for withdrawal, take place at various time points including alongside decisions about starting HMV [1, 7, 8]. However, recent work mapping UK respiratory clinical care using surveys of healthcare professionals working in MND reported that end-of-life respiratory care was most commonly discussed when the patient initiated it (69%) and when there is increased dependence on the ventilator (65%). 37% of the healthcare professional participants reflected that these discussions occurred too late [9].

There is a small body of work that has explored decisions to start ventilation [10–13] and transitions from non-invasive to invasive ventilation [14–16]. Studies exploring plwMND’s decisions to start HMV report findings around the significance of the intervention alongside the patient’s perspective on the prolongation of life, fear of the intervention, and information and knowledge about HMV [10, 13, 17]. All studies highlight the autonomous focus on patient decision-making, Greenaway et al. [10] also illustrate a theme of family and healthcare professional support, or pressure, impacting patient decision-making. Notably, the patient’s knowledge that they can discontinue HMV is only cited as a theme in the paper from Young et al. [12].

Little is known about the extent or nature of end-of-life discussions between patients, families, and healthcare professionals, what informs decisions, or how patients and families are supported through the experience [18]. While some patients who are dependent on HMV wish to remain so until their death, a proportion wish to stop their HMV sooner [19–22]. Some may have made a valid and applicable advance decision to refuse treatment (ADRT). In the UK clinical knowledge suggests a lack of understanding about the processes and legal issues related to discontinuing HMV [23]. Professional guidance notes the general lack of published research evidence in this field and a need for further exploration of the issues [7].

Evidence about decision-making suggests that relational autonomy is often a central facet as patients function in the context of social and family histories that cause them and their families to have ‘overlapping considerations’ and ‘intertwined lives’ [24]. Patients ultimately make decisions about commencing HMV but these are rarely made in isolation or without consideration for their families [10, 11]. However, little is known about decision-making regarding HMV use at the end-of-life, once a plwMND is dependent on HMV, or ‘fully ventilated’ [8, 18]. This study explored this end-of-life decision-making about HMV use from the perspectives of plwMND, family members, and bereaved family members.

## Methods

### Study design

This UK-wide, qualitative interview study was designed to explore end-of-life decision-making about HMV use in MND from the perspectives of plwMND, family members, and bereaved family members. Drawing on an interpretive constructivist methodology, the research aims to provide insights into the diverse ways individuals or groups understand, experience, and navigate health and illness within their social and cultural contexts [25]. A flexible approach to qualitative semi-structured interviews was employed to accommodate the needs of plwMND and their family members, including the bereaved [25, 26]. PlwMND may struggle to communicate due to dysarthria, difficulty projecting their voice, or by speaking whilst using HMV. They also may fatigue easily. Family members, including the bereaved, are often emotionally and physically exhausted so it was vital to adapt interviews to accommodate participants’ needs [26–28].

### Eligibility

#### Bereaved family members

Participants were eligible if they had experienced bereavement after being involved in the care of someone dependent on HMV to sustain their breathing. Bereaved family members were recruited if they had either experience of the person with MND dying following the withdrawal of their HMV, or when the person with MND had died with their HMV in place. They could not take part in the study until at least eight weeks after their bereavement, there was no upper time limit.

#### People living with MND (plwMND) and family members

PlwMND were eligible if they were considered dependent on their HMV. This definition varied across the UK. Flexibility was employed with focus on patients using HMV for 16 h or more per day who were willing to talk about their experiences, goals and values for future care. Family members could, but did not need to be, from the same household as the plwMND. They were eligible if they were currently involved in supporting someone with MND who was dependent on their HMV.

### Recruitment

Participants were either approached by a clinician involved in their care, or, self-referred to the study. The clinicians involved in the study were either part of the project advisory group established at the funding application stage or recruited using a snowball effect from this network and study publicity. Approvals were then sought from each of the relevant NHS Trusts or hospices. If approached by a clinician, potential participant were provided with the Participant Information Sheet. Individuals could contact the researcher [EW] directly for more information or to take part. Study information was shared on social media, via patient and family support networks, the Motor Neurone Disease Association website and branch newsletters, as well as the MND Clinical Studies Group. There was also a study-specific website. Those self-referring to the study did so by contacting the researcher via the contact details provided. They were also sent the Participant Information Sheet via email or post and any queries were answered before setting-up a suitable time for an interview.

### Data collection

Interviews were conducted by EW between May 2021 and December 2023 via a video platform, telephone, email, or in-person. Before the interview, consent was collected, primarily online, via Microsoft Forms©. However, a small number of participants chose to provide written consent, which was scanned and stored securely. Three people chose to have their verbal consent audio recorded. With permission, all interviews were audio recorded and in two instances video recorded via the online platform to aid transcription and clarity of those speaking with HMV in place. Interview guides were developed for each participant group and tailored to the individual. Notes were written after each interview and occasionally some brief questions were clarified or asked by email follow-up.

Great care was taken during the interview process to create a positive experience for participants. Participants were aware they could pause or stop the interview at any time, although none chose to do this. Whilst some did get upset, they noted this was due to their experiences rather than the exploration of these in the interview process. The participant information sheet signposted participants to a range of support should it be required.

### Analysis

A professional company undertook transcription verbatim. After transcription and anonymisation, all interviews were stored in NVivo14© to facilitate a thematic analysis of the data [29]. Throughout the study EW has drawn on a constant comparison approach to support the interpretation of the data [30]. After each interview, summary notes were written and time was taken to reflect on the interview to identify ways to enhance questions and identify any areas that might require follow-up. Once transcribed - or compiled into a word document for email interviews - interviews were read and anonymised, time was taken to reflect on the process and initial open coding undertaken. While the amount of data and level of co-construction [25] varied depending on the participant and collection method, we feel the relevance, importance and richness of the data did not. Hence all data were analysed in the same way by creating nodes and sub-nodes in Nvivo.

To support this iterative analysis process members of the Project Advisory Groups read and commented on 10 (19%) of the transcripts and findings have been presented, discussed, and revised at a number of the Group’s meetings. Initial codes were then reviewed, refined, and revised through a process of reading and re-reading both transcripts and coded nodes to interpret meaning [31, 32]. Data sets were analysed separately and then together to compare themes across the participant groups [24]. Throughout the process written summaries, memos and diagrams were used to organise and identify links between codes and themes, and across data sets [30, 31]. Pseudonyms have been used throughout along with classification as either ‘plwMND’, ‘family members’ and ‘bereaved family members’. Family dyads are linked together where appropriate.

## Results

Sixty-two individuals took part in 53 interviews for the study. The study included people living with MND (*n* = 16), their family members (*n* = 10), and bereaved family members (*n* = 36). Most were recruited from across seven NHS Trusts and two hospices. Across all groups thirteen participants self-referred to the study. Nine of the patients and family members chose to be interviewed together. This was often essential to facilitate the interview as the plwMND would not have been able to participate without this support. Two of these family members also took part in their own separate interview. Three of the bereaved family member interviews were joint interviews with another bereaved family member from the same family. Interviews took place via video platform (*n* = 31), telephone (*n* = 12), in-person at the participant’s home (*n* = 7), or via email (*n* = 3).

People living with MND and using HMV were predominantly male, family members and bereaved family members were predominantly female (see Tables 1 and 2). As Table 2 shows, all family members who spoke about a plwMND were spouses. For the bereaved family members, the relationship to the deceased person were predominantly spouses (*n* = 26/36), but there were also eight adult children and two parents that took part. All patients had or were using mask ventilation, except two (1 invasive ventilation, 1 Cuirass ventilation). Bereaved family members talked about 15 pwMND who died with their HMV in place and 17 who died after their HMV was withdrawn (*n* = 32).

**Table 1:** Bereaved family member demographics

Bereaved family member demographics *n* = 36		
Gender	Female	30
	Male	6
Age range	28–78 years	
Relationship to the patient	Wife	23
	Husband	3
	Daughter	6
	Son	2
	Parent	2
Time from diagnosis to death	2 months to 8 years*	
Time from death to interview date	9 weeks – 5.5 years	
Place of death (n = 32 individual patients)	Home	19
	Hospice	10
	Hospital	3

**Table 2:** plwMND and family member demographics

	PlwMND *n* = 16	Family members *n* = 10
Relationship status	Married	Married
Gender	Female *n* = 3 Male *n* = 13	Female *n* = 8 Male *n* = 2
Age range	50–73 years	45–71 years
Time since diagnosis	1–9 years*	

*These are approximate. Date of diagnosis was not routinely collected as some participants experienced this as a process rather than specific date

The study generated data with intricate stories spanning a range of topics. Data sets were coded independently, cross-data comparisons then highlighted that the data sets shared key themes with no substantial differences between these themes. However, these themes are viewed from different perspectives, or lenses, depending on participant group. This paper presents the findings from across the participant groups in four interlinked themes: selective decisions, timely decisions, defaulted decisions, and proactive decisions.

### Selective decisions

Despite the nature of the study, some participants did not want to talk to the researcher about end-of-life decisions. Bereaved family members also reflected on how their loved one had avoided or refused to talk about their end-of-life.

*Because he didn’t really want to talk about it. …I had to talk to him about it really, because he didn’t want to talk about it to them [the MND team]. …but I mean would it do you any good? That sort of talk?* (Connie, bereaved family member)

Some also noted that, at the time, they too had not wished to talk about the end-of-life, whereas others explained attempts to encouraged loved ones to engage with this topic. Anna illustrates this in the second quote below. As Dave and Susan reflect, expressing avoidance was not always overt, but often took the form of evading interview questions about the end-of-life:

*Susan: I don’t like talking about negative things.*

*Dave: I think because, well I think [she] has a pretty good quality of life all things considered it probably isn’t something that’s really on, you know, the talking list at the moment.* (Susan (plwMND) and her husband Dave).

However, often when participants indicated that they did not want to focus on talking about end-of-life care or planning, they would then go on to talk about some selective decision they had made. These tended to focus on aspects of what might be termed ‘personal planning’. This was often practical, and emotional preparatory work that patients and families undertook for themselves. This would include things such as making adaptations/improvements to the home such as having a wet room installed, gardening or decorating, clearing clutter, will writing, and funeral planning. These are not necessarily health or care-related plans and some can be achieved without the input of healthcare professionals.

*We’ve got lasting power of attorney with [Tim’s] speech getting worse and stuff, so we’ve done that. We’ve done a will, but we haven’t talked about anything else.* (Anna (family member), Tim’s wife)

For those participants who did not want to engage with end-of-life discussions, they would explain this with the caveat that they preferred to ‘live for the day’, and ‘take each day as it comes’. This indicates those living with MND live with considerable uncertainty, limiting their ability and desire to predict and make decisions about their future care.

### Timely decisions

In living with this uncertainty, patients often noted how challenging it was to theorise about what they might want in the future:

*Now, five years ago, if you’d asked me how I would feel about the position I’m in now, I’d probably say, oh God, I couldn’t live with that. But I can live with that and I can enjoy life with that. So, where will I be in six months’ time? I don’t know. But what I think I have learnt is don’t make presumptions about what you can and can’t live with and still enjoy life. So, I don’t want to make decisions like that now, because when the time comes I might feel quite different about it.* (Phil, plwMND)

*We weren’t at the stage where we needed to make those end-of-life decisions, because we’re not at the end-of-life, and when you get to the end-of-life, I know some people like to think far ahead, but life changes, and people change and that can happen very quickly. So you have to make those decisions at the time that it’s happening, because then it becomes natural and it becomes a natural next step. You make those decisions too early on, it’s a huge leap.* (Sophie (family member), Noah’s wife)

This uncertainty about what may feel the right decision in the future was sometimes challenged by the rapid pace at which things could change. As Thomas notes in his email correspondence, in a period of just a few months he needed to shift his thinking from making some decisions about where he might like to be cared for in the future to actively planning the withdrawal of his HMV.

*March22 - I think I have a pretty good understanding of what will happen when I’m approaching the end. Whilst I’m happy to be at home, we actually think I might get more timely and appropriate care if I’m in the local hospice, and I’ve made this clear.*

*May22 - All of a sudden I’m now on NIV for 20 + hours per day, and I’m thinking about asking for ventilation to be withdrawn sometime during this summer. I’ve only discussed this with my wife [name] at this stage, but I’ve drafted [a letter] to give to my palliative care nurse when I see her next*. (Thomas, plwMND)

Some participants appeared to be, or recalled waiting for a healthcare professional to raise choices about HMV with them. Others were happy to be guided by professionals at the appropriate time. Accepting phrases like ‘*what will be will be*’ (Christine, plwMND) and ‘*it is what it is*’ (Joanne, plwMND) illustrated recognition of the unpredictability of the condition. Timely decisions were therefore those made at an opportune or favourable time and were often prompted by changes in the plwMND’s condition. However, many showed reluctance to formalise their decisions, few had an Advance Decision to Refuse Treatment (ADRT), and the potential for loss of capacity was not well recognised. When asked about making a written plan of his decisions in case he was unable to express them himself, Frank responded:

*Well that’s not something that’s likely to occur with MND is it, because MND does not affect my mental capability* (Frank, plwMND).

There was also evidence from the data that some participants did not have a clear understanding of what might happen during the dying process, what might be available to them, or what options they might have at this time. This limited, or mis-understanding, of the dying process restricted the kinds of decisions that could be made in advance.

*I probably didn’t ask enough questions myself, because I do remember thinking, I can’t work out what you actually die from with this disease [when you have ventilation]…. So that’s probably my, whether additional information there would have helped. …at what stage do you get to that it’s no longer working, or I don’t think I’d clicked that actually it could no longer work.* (Jacky, bereaved family member)

*It would be interesting to know the process of it that it would take. …I’m a bit of a coward when it comes to pain. Just out of interest, because no one has discussed that with me. When I started the consultations they wanted to tell me all about end-of-life, and we decided that we weren’t at the stage where we needed to know such things, and that we would just take life a day at a time. …but it wouldn’t hurt now to know is it just a question of switching the machine off, or are there any medications that I might have?* (Noah, plwMND)

Some respondents referred to having been told they could ‘stop any time’, often in early consultations about starting HMV. However, this did not always seem to be equated with the option to withdraw HMV at the end-of-life. Others felt that discussions placed too much emphasis on the option of withdrawal, with limited discussion about what might happen if they decided to continue with HMV:

*I would quite like to know actually, yeah I would quite like to know. So yeah, and I guess maybe when we get to the point where he can’t tolerate any higher pressures then that is a question that I would ask, but it certainly hasn’t been offered. The only thing when I had that initial conversation with the respiratory physio … So that’s the only information that we’ve had about end-of-life, apart from we are always told you can turn it off if you want to.* (Sophie (family member), Noah’s wife)

In their separate interviews, Noah and Sophie, illustrate how patients and family may have different information needs. This could be especially relevant for family members, as the possibility of rapid changes might lead to a situation where the patient was unable to engage in discussions about their preferences before more timely conversations take place:

*With my mum it all happened quite quickly with her breathing and [the MND nurse] said that she hadn’t had chance to have that conversation with her because it literally went from zero to a hundred very quick and I hadn’t spoke to her. I do always know that my mum has always said that if you could go to Switzerland*^1^*and do whatever that wouldn’t be for her, she was in that mindset, so I always thought she would have said no to [withdrawal of her ventilation] anyway.* (Victoria, bereaved family member)

Like Victoria, a few participants aligned the withdrawal process with assisted dying and euthanasia. There seemed to be a limited understanding of the process of withdrawal and the UK’s legal position on the right to refuse treatment.

### Defaulted decisions

Across all participant groups there were people who did not want to talk about the future or end-of-life care decisions. Not wanting to talk about end-of-life was often framed as ‘staying positive’ and some reported believing in faith or fate to guide their course. In some instances, it was apparent that family members wanted a clearer understanding of the dying process and to make some plans for the future.

*Anna: I suppose I do worry for the future if you deteriorate more, about how I’ll manage.*

*Tim: I’m not worried.*

*Anna: It’s all right for you.*

*Tim: I know what’s ahead of me* (Tim (plwMND) and his wife Anna (family member)).

For those who became dependent on HMV and did not want to discuss end-of-life, decisions were often defaulted to others. With no legal decision in place, once unable to contribute to decision-making due to loss of capacity, some patients died with the ventilator in place. For others, the ongoing use of HMV was deemed to be clinically futile and withdrawal was implemented in the patient’s best interests. This bereaved family member recounted how this decision-making involved her by default as the next of kin. With the support and guidance of a healthcare professional, she was able to agree on a decision she was comfortable with.

*So the option we were given was, did we want to take the mask off and, not see what happens, they knew what was going to happen, but did we want to do that by taking the mask off or did we want to take the mask off and put his old one on, which might then take a bit longer but all of it’s going to be the same effect, which I couldn’t really decide, but then the consultant said he would suggest just taking it off, because they all knew it as well, [he] hated the mask.* (Jacky, bereaved family member)

Best interests decisions such as these were often based on relative’s knowledge of their loved one, previous conversations on the topic, or some verbally expressed wishes about the type of end-of-life care they might want to receive. For example, they may have expressed a wish not to suffer, not to be in pain, or like Jacky’s husband, a dislike of his mask. This informed the decision, once it was ‘*clear that this was the end*’ and that this latest respiratory decline was ‘*not something he was coming back from’* (Jacky), which allowed some brief time at the end of his life without the mask.

However, clinical decision-making could be more difficult when the patient’s wishes were not expressed, either verbally or via an ADRT and the health professionals attending the patient are not familiar with that person. Lisa explains how her husband had been reluctant to formalise his wishes about his ventilation, despite having made some other decisions (not wanting his ventilation in place and wanting to die at home), and the subsequent impact on his death:

*Early on after his diagnosis the [palliative care] nurse did come round and she did have some papers with the advanced directive forms. And [he] wasn’t interested in completing them at all. So we put that aside and then when he started to get worse in 2017 the subject was broached again and again it was something that he, he did not want to complete those forms. He did express that he wanted to die at home. He did express that he didn’t want to die on the machine. … But he wouldn’t even write a Will*.*… I do remember being quite angry with [out of hours doctors], not verbally angry, but I was angry, but I do understand why. [Husband] was somebody that they’d never met before, he didn’t have an advanced directive, they could only go with what I told them and they did look at the district nursing records as well. …I did have quite a lot of issues with not being able to [withdrawal his ventilation] sooner when I felt that the time was right and that I felt that time was right the night before.* (Lisa, bereaved family member)

This participant’s distress was evident in her interview. Several bereaved family members also recounted having limited understanding of how the ventilator might impact the dying process. They highlighted that HMV appeared to be maintaining breathing function beyond the natural deterioration of other bodily functions. When bereaved family members recognised this, it was generally a source of distress as they considered it prolonging their loved ones’ suffering.

*We were at home alone with him, …I know that your blood pressure drops and your temperature and that, so I’d done all that. So I knew he should have been gone …yet he was still alive. And I said to my daughter is this ventilator keeping him alive? Well not exactly alive, but it’s keeping him breathing … I’m so angry that nobody told me that, because that man could have possibly not been suffering for so many days if somebody had explained that to me.* (Grace, bereaved family member)

It is at these moments that recognition of the clinical futility of the HMV was reported to be important. Narratives from bereaved family members suggested that if health professionals recognised that the patient had reached the point of dying and that maintaining HMV was no longer the best course of action, the treatment could be withdrawn, as not doing so would prolong dying. Once recognised, almost all families in this situation reported agreeing with health professionals or initiated this suggestion themselves.

*The lead nurse from the [local] Hospice …she said that I think it’s time now …It’s just a machine keeping her alive. So I’m guessing from an ethical perspective that it got to the point where it was the right thing to do and it was all explained and we just agreed to say we were all ready as well, because it’s just, enough is enough really* (Owen, bereaved family member).

While Lasting Power of Attorney (LPA)^2^ was not asked about directly, five participants mentioned having discussed knowledge of this legal requirement for making health and care decisions on behalf of their loved one. Three participants (one bereaved family member and two current family members) noted that they had this document in place.

### Proactive decisions

Few had written ADRTs or a formal statement about their wishes about the HMV element of their care, but more had expressed wishes about other aspects, as illustrated by Lisa’s quote above. Place of care/death was most commonly cited as a decision that had been discussed with planning in place to achieve this. For example, a few plwMND had visited their local hospice with plans to move there for their final days, others reported discussing how they might be supported to stay at home. Of the few that did have an ADRT in place an example of the wording, read by the patient’s bereaved wife is below:

*There was also this advanced decision to refuse treatment. …So he refused resuscitation under any circumstances and he refused invasive ventilation and tracheostomy ventilation. And then the withdrawal of it. So then* “Please withdraw my non-invasive ventilation when I am unable to communicate. My hands are functionless, I cannot write. …When I lose the ability to speak verbally or to use an electronic communication aid or to reliably answer yes or no to simple questions”. *So I mean he couldn’t physically sign it but it was all done and we had a witness came and went through it all. And also that’s when he did the DNR as well.* (Amy, bereaved family member)

Another plwMND also spent time with one of his lead nurses to draft his statement of wishes with a specific mention of what to do if he died at home with his ventilator in place. This wife recounts the importance of making this decision and the support provided by the nurse during this process:

*One of the discussions was about taking the mask off, taking the ventilator off. And if he died at home to write in that to give permission for me to turn the ventilator off, because as she was explaining he stops breathing but the ventilator still works and that is not, and he could be waiting a while for someone to come at home and that’s not, you don’t necessarily want to be sat here with your husband and the ventilator’s going and they’re dead. … He said I don’t want you to have to do that, I don’t want you to have to do that. I said “Love, I’d rather do that than the other. I’d rather see your face properly.” But it was difficult decisions, but they were handled really well by [ventilation nurse] and we got through that.* (Kate, bereaved family member)

The first individuals plwMND discussed their decisions with were often family members. Some were formalised in writing, but it was not always clear if or how much had been communicated to professionals. Some explained that decisions could not be based solely on their assessment of their own quality of life, but were also guided by those around them. In proactive decision-making family members reported the decision to be the patient’s, but that they supported the decision.

*Let’s say if my [operation for tracheostomy ventilation were cancelled] because it’s too late to operate in my condition, I would have to take stock and reassess my situation. These thoughts are never personal, they based on the effect and burden on your loved ones and other people*. (Paul, plwMND)

*Yes, so I guess we were involved in it, but it was more a case of she knew what she wanted and we very much respected it. Yeah we were very much like well we can’t influence you in it.* (Sam, bereaved family member)

For those patients who were keen to make decisions about their future care, this became a source of comfort to them, to feel that they may have some assurances about how they might die and a chance to be a conductor in their own death. As noted above, a number of people may have initially been unclear about the process of withdrawal of HMV or even that it was a legal option for them. When asked about the importance of having the choices at the end of his life Geoff stated:

*I didn’t realise they existed. Having realised they existed it’s empowering ...It enabled me to have control, it enabled me to make a decision. It gave my family the opportunity to move on. It allowed me to have the timescale to tidy up my business ready for handing it on [to someone else], and it gave me the summer. Because I was a keen gardener, I enjoy being in the garden. … Because I do not want to die, but I don’t want to live the life that I have now.* (Geoff, plwMND)

Those that had made proactive plans for the withdrawal of their treatment used words like ‘empowering’, ‘control’, and ‘choice’.

*We had that control, we know that’s what happened and be in control of it and it was like, ultimately he died of Motor Neurone disease, but it was us that made that decision of when that happened.* (Julia, bereaved family member)

*In one sense mum felt very much like she had control back in her hands, the disease robbed her of control over every aspect of her life but not decision making, and she made that decision [to withdraw her ventilation] and that was entirely her decision and she was entirely in control of it.* (Olivia, bereaved family member)

Some participants recognised that becoming dependent on their ventilator empowered them to die at a time of their choosing by declining further HMV. Sam describes his mother’s “privilege” of having a novel control over her dying, that only existed because she chose to start NIV:

*…she just wanted to do it on her own terms, which I think is actually a real privilege in a way, but a lot of conditions and sometimes MND don’t have that control, I guess, and it’s not obviously euthanasia but it is almost felt like, a little bit like that she did then have control over it. And it was almost like that she hadn’t had control over it the whole time and then by having that it was almost like a two fingers up to it, which I think she quite liked: ‘yeah well I’m going to do it on my own terms, not when you want me to go’.* (Sam, bereaved family member)

*I want to be in control of my ventilator and to decide when I don’t want to use it anymore. I don’t know when that will be, but I’ll know when I get there! I guess I see it as a possible exit route from MND.* (Thomas, plwMND)

Some patients made proactive decisions about their death. This enabled an active celebration of their life and reassured families that their wishes had been met.

*He’d planned it all and he said will you come at four o’clock? And it was just [son], his elder brother, and [wife] and us. …I just heard him say ‘I’ve had a wonderful life, I’m at peace, I’m ready to go’. …once we’d heard him say that so loud and clear, everybody felt they could relax a bit almost and just fully engage with [plwMND]’s wishes and embrace what was happening and it really was beautiful* (David, bereaved family member).

Patients reported their reasons for the timing of their withdrawal. These included time of year, such as wanting to see another summer; wanting it to be during the school holidays so that grandchildren could be supported; having time to put personal and/or business affairs in order; wanting to participate in key events such as a wedding, or to avoid key dates such as birthdays. Primarily timing was based on the patient’s feeling that they had ‘had enough’. As Geoff (plwMND) above notes at the end of his comment, and as Lauren (bereaved family member) stated ‘*living might be too much but that doesn’t mean you want to die’*.

## Discussion

This paper has presented findings from a study to explore end-of-life decision-making about HMV in MND. The perspectives of plwMND (*n* = 16), their family members (*n* = 10), and bereaved family members (*n* = 36) were gathered over a period of two and half years. The process of extensive qualitative analysis has drawn out four core themes from across all participant groups: selective decisions, timely decisions, defaulted decisions, and proactive decisions. Weisman [33] suggests that peoples’ dying reflects the way that they have lived, and is the foundation for person-centred palliative care [34]. This personalisation is shown in this paper. The data shows that decision-making is complex, is usually evolving, and rarely entirely autonomous [10]. Cognition was not assessed for participation in this study, however clinicians need to recognise the potential for impact on patient decision-making. Decisions about the use of HMV at the end-of-life shared by our participants took account of quality of life, symptom control, impact on families, faith, pets, layouts of houses, services available and much more.

This study shows that, even when plwMND and their family members are reluctant to make specific decisions about the end-of-life, they are willing to select certain aspects of personal planning to enact. As noted by Pollock et al. [35], families tend to focus on aspects of their personal lives such as making a will, gifting items to relatives, and making burial or funeral plans. Advance Care Planning is standardised in UK national policy [36], but we continue to see patients not wanting to engage with the formal written elements of this process which facilitate enactment of their wishes [37–42]. Being unwilling to discuss end-of-life care as part of the research may suggest that these participants had not had these discussions with healthcare professionals. Even for those that had discussed certain elements with family members, it was not always clear if, or how, this information had been shared with professionals involved in their care. Withdrawal of HMV can be practically, emotionally, and ethically challenging for healthcare professionals [23, 43]. Without formal recording of decisions, some healthcare professionals may be reluctant to stop HMV at the end-of-life, as illustrated by Lisa’s narrative of her husband’s death.

The reluctance to create written documentation was often driven by the uncertainty of the condition [10]. Thorborg et al. [16] also identified this in their study of patients’ decisions on whether to choose invasive HMV or not. They use the term ‘so far’ from their data to illustrate that timely decisions are decisions ‘for now’ and may need to be changed in the future [16]. Greenaway et al. [10] also identified the concept of time in their interview findings with patients making decisions about starting NIV. As illustrated in the presented study, Greenaway et al. [10] note timeliness as influential to patient decision-making with concepts around living in the moment, what they refer to as ‘right thing, right time’ (also see [11]) and predicting the future, or what we have termed living with uncertainty. However, delaying or not wanting to make decisions can also be due to a lack of information. Some participants in our study suggested that they did not have a clear understanding of what support and options might be available to them as they approached the end of their life, and what the process of dying, with or without ventilation, would be like. This may have hindered advance decision-making resulting in late or even defaulted decisions. It was apparent from bereaved family member accounts that the impacts of decisions, or lack thereof, could have implications for bereavement.

Often patients do take a more passive approach to decision-making and family members act as surrogate decision-makers [44, 45]. In the UK however, family members can only *inform* a decision in someone’s best interests but are not the decision *makers* unless there is a LPA in place. It was not clear from this study if or how families understood this. The role of family members in decision-making and concerns about how much they are aligned with those of the patient is highlighted in Symmons et al.’s [45] literature review. In this study, many discussed thoughts on withdrawal of treatment more generally and shared the wishes of their loved one by describing desired outcomes such as avoiding pain, avoiding suffering, or wanting to be in the place of their choosing. Romo et al. [46] posit that, while not actively making decisions, in this way patients are arming relatives with the tools they need to inform appropriate decisions on their behalf. In their study of older people living at home in the USA, the authors suggest this is a way for dying people to achieve a sense of control without proactively taking control of decisions. Moreover, evidence suggests patients want family members to make decisions on their behalf when they cannot and allow leeway in how these decisions are made [44]. For participants in this study, family members were primarily spouses or a close relative. All had been involved in the care for a long time. No one noted any familial discord about end-of-life decisions made and many recounted family support and agreement with decisions about HMV.

The evidence and guidance available suggest discussions about stopping ventilation should take place early, even at initiation [7, 9]. This study has illustrated some lack of clear understanding of the dying process, the UK’s legal position and the options available at the end-of-life, echoing Phelps et al. whose work specifically focused on withdrawal of ventilation [43]. For some, this appeared to hinder decision-making. This has significant impact for health professionals delivering care and suggests that discussions need to go beyond information giving at specific time points [9]. Interwoven with this, findings also suggest that there may be reluctance to discuss end of life management of HMV, often until the patient recognises the timeliness of the discussion based on their prognostic awareness [47]. Families may require an iterative process to deepen their understanding of the illness trajectory so that cognitive and emotional coping strategies can be developed [47]. This greater understanding of the complexity of decision-making for those with MND and life sustaining treatments can support clinicians in developing ways to assess patient values to help understand how they can best align decision-making discussions with patient preferences and stage of prognostic awareness.

The use of HMV at the end-of-life for plwMND poses a unique position for decision-making as some participants noted. Proactive decisions about HMV centred on patients’ wishes to have HMV withdrawn once they were no longer able to contribute to a decision and the treatment was recognised as no longer in their best interests. Some had written ADRTs which, according to plwMND, contained similar content to that of the one recounted by Amy. Patients also discussed their reasons for their choice of timing for the withdrawal. These findings support those of ven Eenennaam et al. [48] who explored perspectives on decision-making regarding gastrostomy for people with MND in the Netherlands. The authors refer to what they term ‘control in the absence of choice’, patients felt forced to have the gastrostomy due to the progression of the disease but felt they retained some control over the timing of this [48]. This parallels the expressions by participants in this study to have control over the timing of their death. Participants appreciated that being dependent on the ventilator gave them the choice to decide when to die. Ventilation is often promoted as reducing symptoms and extending life. This study has identified an additional benefit of HMV. Some patients appreciated that once dependent, choices about withdrawal of HMV gave them greater control over when, and how, they die. We anticipate this becoming an ever more important concept as increasing numbers of patients select life-extending technology and can decide to stop using that technology.

### Strengths and limitations

The study used a necessarily flexible approach to interviews in order to accommodate the communication issues experienced by plwMND who are dependent on their ventilation. The flexible approach to data collection allowed participants to take control of how and when they wanted to participate. We would suggest that such flexibility was a strength of the study and should be embodied in studies with populations where there are significant barriers to research participation, such as communication. This flexibility extended beyond offering a variety of ways of participating and included adapting interview guides and the way that questions were phrased. Sometimes it was necessary to use more closed questioning and shorter interviews to accommodate participant fatigue and their communication preferences. We recognise that this expanded approach to conventional qualitative interviews has impact for the nature of the data collected, however we do not feel this has affected the credibility of the data [49]. Further discussion of these expanded interview techniques can be found in our recent publication [50].

Dying is such an individual event that it was important to avoid the risk of falsely assuming premature coding stability, making it a significant strength to be able recruit a large range of participants, including patients themselves. The study has a high proportion of female caregivers across the data sets. We suggest this is in line with the population of plwMND, particularly those who choose more complex interventions such as ventilation [3, 10, 48]. Given the nature of the study and the small population of potential participants, it was not feasible to sample for diversity in any way. As such, data were not gathered on ethnicity or religion, although a small number of participants did comment on their religious beliefs. It is recognised that this may have impact on the range of responses about culturally diverse decision-making values. We must also acknowledge the challenge of recruiting current family members to the study. This was likely due to caring responsibilities. As such, the majority (*n*=7/10) took part in joint interviews as a patient–family member dyad, which may have affected their responses. The three family members who were able to take part individually noted that this was possible as they had homecare workers in place at the time of the interview and were able to leave the plwMND to take part. Time since bereavement ranged from nine weeks to over five years. This could have implications for the recall of specific details such as dates and the re-telling of experiences.

## Conclusion

This study gathered data from people with experience of HMV dependence, and is strengthened by the large sample size for this population and sensitivity of the topic. Findings ensure a better understanding of patient and family approaches to decision-making using the four themes and legal provisions. Due to the uncertainty faced by those with MND, participants who were unwilling to discuss dying often preferred to make decisions in a timely manner, or default these decisions to others. However, some did and wanted to make choices about their end-of-life. Proactive decisions, including advance care planning and withdrawal of treatment, were found to empower patients, providing a sense of control over the timing of their death. A significant portion lacked a clear understanding of the dying process and available options. The study highlights the complex and varied nature of end-of-life decision-making in the context of ventilation technology and MND and the important role healthcare professionals can play in giving information, guidance and support in a personalised and sensitive way.

### Electronic supplementary material

Below is the link to the electronic supplementary material.

Supplementary Material 1

Supplementary Material 2

Supplementary Material 3

## Data Availability

The datasets generated and/or analysed during the current study are not publicly available. Data sharing was not included in the ethical approval as the study involves personal accounts of patients’ and families’ experiences. It is essential that we maintain confidentiality and anonymity but sufficiently anonymised parts of the data are available from the corresponding author on reasonable request.

## Footnotes

1 Switzerland, along with a number of other countries has a legalised form of Assisted Dying that permits an individual to assist in another’s suicide. This permits doctors to prescribe and administer medications which would end a person’s life. This is not legal in the UK. The euphemism ‘going to Switzerland’ was regularly used to express views about choices to end one’s life.

2 In the UK, a LPA is a legal document that appoints someone of the patient’s choice to manage (1) health and welfare, and/ or (2) property and financial affairs, if they are no longer able to manage those decisions for themselves.

## References

[1] Leigh PN, Abrahams S, Al-Chalabi A, Ampong M-A, Goldstein LH, Johnson J (2003). The management of motor neurone disease. J Neurol Neurosurg Psychiatry.

[2] Bourke SC, Tomlinson M, Williams TL, Bullock RE, Shaw PJ, Gibson GJ (2006). Effects of non-invasive ventilation on survival and quality of life in patients with amyotrophic lateral sclerosis: a randomised controlled trial. Lancet Neurol.

[3] Palmer J, Messer B, Ramsay M. Tracheostomy ventilation in motor neurone disease: a snapshot of UK practice. Amyotroph Lateral Scler Frontotemporal Degeneration. 2020;6(52). 10.1183/23120541.RFMVC-2020.52. 10.1080/21678421.2021.1916534 33969757

[4] Schwarz EI, Mackie M, Weston N, Tincknell L, Beghal G, Cheng MCF, Ramsay M, Suh E-S, Kaltsak G, Pattani H, Marino P, Murphy PB, Hart N, Steier J. Time-to-death in chronic respiratory failure on home mechanical ventilation: a cohort study. Respir Med. 2020;162(105877). 10.1016/j.rmed.2020.105877. 10.1016/j.rmed.2020.105877 32056675

[5] Masrori P, Van Damme P (2020). Amyotrophic lateral sclerosis: a clinical review. Eur J Neurol.

[6] National Institute of Health and Care Excellence (NICE). Motor neurone disease: assessment and management. 2016. Overview | motor neurone disease: assessment and management | Guidance | NICE.

[7] Association for Palliative Medicine of Great Britain and Ireland (AMP). Withdrawal of assisted ventilation at the request of a patient with Motor Neurone Disease: Guidance for professionals. Guidance for professionals. Fareham: Association for Palliative Medicine of Great Britain and Ireland (AMP); 2015 APM-Guidance-on-Withdrawal-of-Assisted-Ventilation-Consultation-1st-May-2015.pdf.

[8] Motor Neurone Disease Association. Ventilation for motor neurone disease – 8B 2022; 8B - Ventilation for motor neurone disease (mndassociation.org).

[9] Musson LS, Baxter SK, Norman P, O’Brien D, Elliott M, Bianchi S (2022). Delivery of non-invasive ventilation to people living with motor neuron disease in the UK. ERJ Open Res.

[10] Greenaway LP, Martin NH, Lawrence V, Janssen A, Al-Chalabi A, Leigh PN (2015). Accepting or declining non-invasive ventilation or gastrostomy in amyotrophic lateral sclerosis: patients’ perspectives. J Neurol.

[11] Martin NH, Lawrence V, Murray J, Janssen A, Higginson I, Lyall R (2016). Decision making about Gastrostomy and noninvasive ventilation in amyotrophic lateral sclerosis. Qual Health Res.

[12] Young J, Marshall C, Anderson E (1994). Amyotrophic lateral sclerosis patients’ perspectives on use of mechanical ventilation. Health Soc Work.

[13] Lemoignan J, Ells C (2010). Amyotrophic lateral sclerosis and assisted ventilation: how patients decide. Palliat Support Care.

[14] Kurisaki R, Yamashita S, Sakamoto T, Maruyoshi N, Uekawa K, Uchino M (2014). Decision making of amyotrophic lateral sclerosis patients on noninvasive ventilation to receive tracheostomy positive pressure ventilation. Clin Neurol Neurosurg.

[15] Kawamata J, Yamamoto D, Matsushita T, Matsumura A, Suzuki S, Hisahara S (2017). Decision making of using invasive mechanical ventilation in patients with amyotrophic lateral sclerosis in Sapporo. J Neurol Sci.

[16] Thorborg T, Finderup J, Winther DS, Lorenzen CK, Dreyer P (2023). The experiences of patients with amyotrophic lateral sclerosis of their decision-making processes to invasive home mechanical ventilation—A qualitative study. Nurs Open.

[17] Martin NH, Landau S, Janssen A, Lyall R, Higginson I, Burman R (2014). Psychological as well as illness factors influence acceptance of non-invasive ventilation (NIV) and gastrostomy in amyotrophic lateral sclerosis (ALS): a prospective population study. Amyotroph Lateral Scler Frontotemporal Degeneration.

[18] Wilson E, Lee J-S, Wenzel D, Faull C (2022). The use of mechanical ventilation support at the end of life in motor neurone Disease/Amyotrophic lateral sclerosis: a scoping review. Brain Sci.

[19] Baxter S, Baird W, Thompson S, Bianchi S, Walters S, Lee E (2013). The use of non-invasive ventilation at end of life in patients with motor neurone disease: a qualitative exploration of family carer and health professional experiences. Palliat Med.

[20] Oliver DJ, Turner MR (2010). Some difficult decisions in ALS/MND. Amyotroph Lateral Scler.

[21] Messer B, Armstrong A, Doris T, Williams T (2020). Requested withdrawal of mechanical ventilation in six patients with motor neuron disease. BMJ Supportive Palliat Care.

[22] Faull C, Wenzel D (2020). Mechanical ventilation withdrawal in motor neuron disease: an evaluation of practice. BMJ Supportive Palliat Care.

[23] Phelps K, Regen E, Oliver D, McDermott C, Faull C (2017). Withdrawal of ventilation at the patient’s request in MND: a retrospective exploration of the ethical and legal issues that have arisen for doctors in the UK. BMJ Supportive Palliat Care.

[24] Ho A (2008). Relational autonomy or undue pressure? Family’s role in medical decision-making. Scand J Caring Sci.

[25] Rubin H, Rubin I (2005). Qualitative interviewing: the art of hearing data.

[26] Philpin SM, Jordan SE, Warring J (2005). Giving people a voice: reflections on conducting interviews with participants experiencing communication impairment. J Adv Nurs.

[27] Luck AM, Rose ML (2007). Interviewing people with aphasia: insights into method adjustments from a pilot study. Aphasiology.

[28] Carlsson E, Paterson BL, Scott-Findlay S, Ehnfors M, Ehrenberg A (2007). Methodological issues in interviews involving people with communication impairments after acquired brain damage. Qual Health Res.

[29] Jackson K, Bazeley P (2019). Qualitative data analysis with NVivo.

[30] Charmaz K, Thornberg R (2020). The pursuit of quality in grounded theory. Qualitative Res Psychol.

[31] Bazeley P (2021). Qualitative data analysis: practical strategies.

[32] Kvale S, Brinkmann S, InterViews (2009). Learning the craft of qualitative research interviewing.

[33] Weisman AD (1972). On dying and denying: a psychiatric study of terminality.

[34] Österlind J, Henoch I (2021). The 6S-model for person-centred palliative care: a theoretical framework. Nurs Philos.

[35] Pollock K, Wilson E, Caswell G, Latif A, Caswell A, Avery A, et al. Family and health-care professionals managing medicines for patients with serious and terminal illness at home: a qualitative study. Health Serv Deliv Res. 2021;9(14). 10.3310/hsdr09140. 34410684

[36] Thomas K, Russell S (2023). Advance Care Planning in the United Kingdom – A snapshot from the four UK nations. Zeitschrift für Evidenz Fortbildung Und Qualität Im Gesundheitswesen.

[37] Jimenez G, Tan WS, Virk AK, Low CK, Car J, Ho AHY (2018). Overview of systematic reviews of Advance Care Planning: Summary of evidence and global lessons. J Pain Symptom Manag.

[38] Lovell A, Yates P (2014). Advance Care Planning in palliative care: a systematic literature review of the contextual factors influencing its uptake 2008–2012. Palliat Med.

[39] Lund S, Richardson A, May C (2015). Barriers to Advance Care Planning at the end of life: an explanatory systematic review of implementation studies. PLoS ONE.

[40] Pollock K, Bulli F, Caswell G, Kodba-Čeh H, Lunder U, Miccinesi G (2022). Patient and family caregiver perspectives of Advance Care Planning: qualitative findings from the ACTION Cluster randomised controlled trial of an adapted respecting choices intervention. Mortality.

[41] Rietjens JAC, Sudore RL, Connolly M, van Delden JJ, Drickamer MA, Droger M (2017). Definition and recommendations for advance care planning: an international consensus supported by the European Association for Palliative Care. Lancet Oncol.

[42] Zwakman M, Jabbarian L, van Delden J, van der Heide A, Korfage I, Pollock K (2018). Advance care planning: a systematic review about experiences of patients with a life-threatening or life-limiting illness. Palliat Med.

[43] Phelps K, Regen E, McDermott C, Oliver D, Faull C (2022). Withdrawal of assisted ventilation at the patient’s request in MND: a Retrospective Exploration of the ethical and legal issues concerning relatives, nurses and Allied Health Care professionals. medRxiv.

[44] Vig EK, Taylor JS, Starks H, Hopley EK, Fryer-Edwards K (2006). Beyond Substituted Judgment: how surrogates navigate end-of-life decision-making. J Am Geriatr Soc.

[45] Symmons M, Ryan K, Aoun SM, Selman LE, Davies AN, Cornally N (2023). Decision-making in palliative care: patient and family caregiver concordance and discordance—systematic review and narrative synthesis. BMJ Supportive Palliat Care.

[46] Romo RD, Allison TA, Smith AK, Wallhagen MI (2017). Sense of control in end-of-life decision-making. J Am Geriatr Soc.

[47] Jackson VA, Emanuel L (2023). Navigating and communicating about serious illness and end of life. N Engl J Med.

[48] van Eenennaam RM, Rave N, Kruithof WJ, Kruitwagen-van Reenen ET, van den Berg LH, Visser-Meily JA (2023). Control in the absence of choice: a qualitative study on decision-making about gastrostomy in people with amyotrophic lateral sclerosis, caregivers, and healthcare professionals. PLoS ONE.

[49] Howard F, Crowe S, Beck S, Haljan G (2021). Attending to Methodological Challenges in Qualitative Research to Foster participation of individuals with chronic critical illness and communication impairments. Global Qualitative Nurs Res 8.

[50] Wilson E, Turner N. Expanding qualitative interviewing for studies involving adults with different communication needs: reflections on research with people living with motor neurone disease. International Journal of Qualitative Methods 2024 (in press).

