# Supplementary material for: End of life decision making when home mechanical ventilation is used to sustain breathing in Motor Neurone Disease: patient and family perspectives
Source: BMC Palliat Care. 2024 May 2;23:115. doi: 10.1186/s12904-024-01443-1 (PMC11064348; doi:10.1186/s12904-024-01443-1)
Supplement: Supplementary file 1 — Supplementary Material 1 [file 12904_2024_1443_MOESM1_ESM.docx]

**Exploring end of life decision making with patients with Motor Neurone Disease (MND) using home mechanical ventilation: The perspectives of PwMND.**

**Person with MND interview topic guide**

Introduction

- *Introduction to researcher and study*
- *Interview involves a free and informal discussion; confidential; can stop at any time; no pressure to answer questions/discuss specific topics*
- *Can pause and restart at any time*
- *Permission to record*
- *Completion of consent to interview*

*In this study I am interest in the experiences of people with MND who are using ventilation at home to alleviate the symptoms of MND and can no longer effectively breathe without it (dependent)*

Just to get started can you tell me a bit about yourself?

- Age, employment, family, interests
- Can you tell me about your illness?
- How were you diagnosed? circumstances, symptoms, duration
- How have things been since then?
- Who is involved in supporting you? Family/HCPs?

When did you decide to use ventilation?

- How did you make the decision?
- Who did you discuss it with? Who else involved? Family/HCPs?
- What information were you given?
- Was there any discussion about your use of ventilation in the future?
- Has it been discussed since?
- What do you think about the timings of the discussions you have had?
- Have you used any type of forum/internet group or support group to discuss these issues?

What are your thoughts about the use of ventilation in the future?

- Who have you discussed these with? Family/HCPs/more widely
- Do you have any other wishes for your care in the future?
- How /have you expressed/documented these? Discussed with family?

Do you feel you know enough about what is likely to happen as you become increasingly ill?

- What else would you like to know about? Who to ask?
- Role of family

*Anything else, missed, not discussed?*

*Establish if experiencing any distress as a result of the interview – extend debrief for as long as necessary to re-establish composure*

End of interview and Thanks!
