# Supplementary material for: End of life decision making when home mechanical ventilation is used to sustain breathing in Motor Neurone Disease: patient and family perspectives
Source: BMC Palliat Care. 2024 May 2;23:115. doi: 10.1186/s12904-024-01443-1 (PMC11064348; doi:10.1186/s12904-024-01443-1)
Supplement: Supplementary file 2 — Supplementary Material 2 [file 12904_2024_1443_MOESM2_ESM.docx]

**Exploring end of life decision making with patients with Motor Neurone Disease (MND) using home mechanical ventilation: The perspectives of families.**

**Current Family Member interview topic guide**

Introduction

- *Introduction to researcher and study*
- *Interview involves a free and informal discussion; confidential; can stop at any time; no pressure to answer questions/discuss specific topics*
- *Permission to record*
- *Completion of consent to interview*

*In this study I am interest in the experiences of family members who are involved in the care of someone dependent (using ventilation during the day as well as at night) on home ventilation to alleviate the respiratory symptoms caused by MND.*

First of all: can you tell me a bit about you and your family?

- [name] illness – symptoms, diagnosis, circumstances, duration, location
- relationship with them? involvement in support, care and extent of contact?
- what is it like being involved in caring for someone using HV
- other key people involved – their role

Ventilation:

- how was the decision made to start?
  - - Who/how discussed, information given
    - Was future use also discussed? - thought about that in advance?
- wishes regarding their treatment or care
  - - thoughts about preferences to stop or continue ventilation
    - have these been discussed
    - Agreement/disagreement
    - Does [name] have any other wishes? /wishes for them
- Awareness and communication about dying and anticipated death within the family and between the family and 1. dying person 2. HCPs
- Do you feel you have enough information about what will happen in the future?
  - - Who would you ask for more information?/discuss this with?

The decision:

- reflection on ‘quality’ of decision making: place in decision making process, information, support, family involvement, communication
  - - review of the decision – how/by whom/ recorded
    - positive aspects /negative aspects/what could be done differently
    - what support given/needed
    - concerns for future/fear/worries
    - wider impacts – family/friends/work/social

Key messages:

- for other families/patients/ HCPs – info needed

*Anything else, missed, not discussed?*

*Establish if experiencing any distress as a result of the interview – extend debrief for as long as necessary to re-establish composure*

End of interview and Thanks!
