# Supplementary material for: End of life decision making when home mechanical ventilation is used to sustain breathing in Motor Neurone Disease: patient and family perspectives
Source: BMC Palliat Care. 2024 May 2;23:115. doi: 10.1186/s12904-024-01443-1 (PMC11064348; doi:10.1186/s12904-024-01443-1)
Supplement: Supplementary file 3 — Supplementary Material 3 [file 12904_2024_1443_MOESM3_ESM.docx]

**Exploring end of life decision making with patients with Motor Neurone Disease (MND) using home mechanical ventilation: The perspectives of families.**

**Bereaved Family Member interview topic guide**

Introduction

- *Introduction to researcher and study*
- *Interview involves a free and informal discussion; confidential; can stop at any time; no pressure to answer questions/discuss specific topics*
- *Permission to record*
- *Completion of consent to interview*

*In this study I am interest in the experiences of family members who have experienced bereavement after being involved in the care of someone dependent (using ventilation for 16hrs or more per day) on home ventilation to alleviate the symptoms of MND.*

First of all: can you tell me a bit about yourself and your family?

- [names] illness – symptoms, diagnosis, circumstances, duration, place of care
- relationship with them? involvement in support, care and extent of contact?
- Other key people involved – their role

Ventilation:

- What was it like being involved in the care of someone using home ventilation?
  - - Who else was involved in that care?
- How was the decision made to start?
  - - Who/how discussed, information given
    - When was the role of ventilation in the future discussed? - thought about that in advance?
- Awareness and communication about dying and anticipated death within the family and between the family and 1. dying person 2. HCPs
- wishes regarding their treatment or care
  - - thoughts about preferences to stop or continue ventilation
    - Who did they/[name] talk to?
    - Agreement/disagreement
    - Did [name] have any other wishes? - was it possible to fulfil these wishes?

The last few days:

- reflection on ‘quality’ of experience: medical management, control of symptoms, distress, decision making, family involvement in decision making
  - - positive aspects of the experience
    - negative aspects/regrets – things that might have been otherwise
    - as expected
    - what support given/needed
    - wider impacts – family/friends/work/social

Key messages:

- for other families/patients/ HCPs – info needed

*Anything else, missed, not discussed?*

*Establish if experiencing any distress as a result of the interview – extend debrief for as long as necessary to re-establish composure*

End of interview and Thanks!
